# Pseudohypoaldosteronism type II and sensory neuropathy associated with a heterozygous pathogenic variant in KLHL3 gene, a case report

**DOI:** 10.1016/j.heliyon.2024.e39891

**Published:** 2024-10-29

**Authors:** J.B. Davion, I. Coku, A. Wissocq, A. Genet, J. Poupart, L. Defebvre, V. Huin

**Affiliations:** a Univ. Lille, Inserm, CHU Lille, U1172 - LilNCog (JPARC) - Lille Neuroscience and Cognition, F-59000, Lille, France; b Reference Center for Neuromuscular Diseases, CHU Lille, Pierre Swynghedauw Hospital, F-59000, Lille, France; c CHU Lille, Toxicology and Genopathies Department, UF Neurobiology, F-59000, Lille, France; d Lille Catholic Hospital (GHICL), Neurology Department, F-59462, Lomme, France; e CHU Lille, Neurology and Movement Disorders Department, F-59000, Lille, France

**Keywords:** Pseudohypoaldosteronism, Sensory neuropathy, Mutation, Gordon syndrome, Case report

## Abstract

Pseudohypoaldosteronism type II is a rare Mendelian disorder characterized by hypertension, hyperkalemia, hyperchloremia and metabolic acidosis, despite a normal glomerular filtration rate. Four genes (*KLHL3*, *CUL3*, *WNK1* and *WNK4*) are associated with this disease. Mutations in the *KLHL3* gene cause pseudohypoaldosteronism type II in either an autosomal dominant or a recessive inheritance pattern. Sensory neuropathy has been associated with autosomal recessive mutations in *WNK1*, but not with *KHLH3*. We reported a unique three-generation family with dominant pseudohypoaldosteronism type II and sensory neuropathy. Three affected members of the family underwent neurological examination, nerve conduction studies and exome sequencing. A 13-years-old girl had a history of pseudohypoaldosteronism type II, and suffered from neuropathic pain associated with a sensory neuronopathy. Her mother and grandfather have pseudohypoaldosteronism type II associated with an asymptomatic sensory neuropathy on nerve conduction studies. Exome sequencing revealed in all affected members two missenses at heterozygous state, one pathogenic variant in *KLHL3,* which may be responsible for the sensory neuropathy. This is the first description of neurological features associated with *KLHL3* mutation. Our study expands the genotype-phenotype spectrum of *KLHL3* with the addition of sensory neuronopathy.

## Highlights

• Expansion of the phenotype associated with *KLHL3* mutations.
• Dominant sensory neuropathy associated with dominant PAHII.
• Heterogeneity of the neurological signs.

## Abbreviations:

CUL3: Cullin 3 gene
CMAPs: Compound muscle action potentials
KLHL3: Kelch like family member 3 gene
NCS: Nerve conduction studies
PHAII: Pseudohypoaldosteronism type II
SNAP: Sensory nerve action potential
WNK1: WNK lysine deficient protein kinase 1 gene
WNK4: WNK lysine deficient protein kinase 4 gene

## 1 Introduction

Pseudohypoaldosteronism type II (PHAII) is characterized by hypertension, hyperkalemia, hyperchloremia, and metabolic acidosis, despite a normal glomerular filtration rate. Four genes are associated with PHAII: *WNK1*, *WNK4*, *KLHL3*, and *CUL3* [1]. Mutations in *KLHL3* cause PHAII in either an autosomal dominant or a recessive inheritance pattern. *WNK1* mutations cause an autosomal dominant PHAII, or an autosomal recessive hereditary sensory and autonomic neuropathy type IIA. To date, no neurological damage has been reported with *KLHL3* mutations. We report a unique three-generation family diagnosed with both PHAII and sensory neuropathy.

## 2 Case/case series presentation

The proband, a 13-year-old French girl, came to the Lille University Hospital for excruciating pain in the left heel. She was diagnosed with PHAII at age 11 because of hyperkalemia which we treated with hydrochlorothiazide 12.5 mg/day (started at age 11). She complained of left heel pain that suddenly appeared one morning at age 12 and has persisted since. She described the pain as “stabbing”, being present all day and night (visual analog scale rated 6/10, with paroxysmal episodes rated 9/10), and also worsened by pressure, touch, contact with water (allodynia), and whilst walking. At age 18, the patient complained of neuropathic pain to the left knee. She took the habit of walking on tiptoes on the left side to prevent pain or to use crutches to limit contact with the floor. Treatments for neuropathic pain, such as gabapentin (up to 600mg 3 times/day, tried at age 13), amitriptyline (up to 25 mg/day, tried at age 14), duloxetine (60mg/day, tried at age 14), local lidocaine (700 mg, tried at age 14) and 8 % capsaicin patch (1/day, tried at age 15), were ineffective. Clinical examination revealed normal motor strength of the four limbs, normal deep tendon reflexes, and no signs of upper motor neuron syndrome. Sensory examination only revealed slight hypopallesthesia of the four distal limbs and slight oscillations in the Romberg maneuver. There was a mild distal sensory loss of the left foot and leg, not attributable to a specific nerve territory. Examination of the protopathic, thermic, and analgesic sensitivity was normal, including the painful area. She complained of mild episodes of hand and foot erythrosis and excessive sweating. The left heel was normal. Nerve conduction studies (NCS) was performed on a Dantec® Keypoint® G4 system and showed diffuse reduction of the sensory nerve action potential (SNAP) amplitude of the four limbs in a non-length dependent pattern, with normal compound muscle action potentials (CMAPs) and needle electromyography, thus suggesting sensory neuronopathy (Camdessanché score = 7.6) [2] (Supplementary Table S1.). After a six-year follow-up, the complaints and clinical examination remained stable.

Her mother was diagnosed with PHAII at age 37 because of chronic hyperkalemia and treated with furosemide (40mg/d). She had no neurological complaints and a normal clinical examination. NCS at age 48 showed a bilateral reduction of SNAP amplitude in the lower limbs, normal SNAP amplitudes in the upper limbs, except for the median nerves (related to carpal tunnel syndrome), and normal CMAPs amplitudes for the limbs, except for the left extensor digitorum brevis muscle. Needle electromyography was normal. This profile was suggestive of sensory length-dependent polyneuropathy (Camdessanché score = 3.1) (Supplementary Table S1).

The proband's maternal grandfather had a history of chronic hyperkalemia treated with sodium polystyrene sulfonate (15g/day), without any diagnostic work-up, hypertension treated with perindopril-amlodipine (5/10 mg/day), inguinal hernia surgery, and trigger finger surgery. From the age of 75, he complained of left leg pain. Neurological examination revealed isolated weakness during extension of the left foot, graded 2/5 on the Medical Research Council scale, a protopathic sensory deficit of the left foot. Additionally, he had a mild gait trouble since the age of 70, sensory ataxia on examination and severe bilateral hypopallesthesia of both lower limbs. Deep tendon reflexes were all normal. NCS studies revealed a diffuse reduction of SNAP amplitude for the four limbs in a non-length dependent pattern. CMAPs amplitudes were decreased when recording muscles innervated by the left sciatic nerve (extensor digitorum brevis, tibialis anterior, abductor halluces brevis muscles) on the left side, whereas examination was normal on the right side and for the upper limbs. Needle electromyography revealed a neurogenic pattern in the muscles innervated by the left sciatic nerve (extensor halluces longus, peroneus longus, tibialis anterior, gastrocnemius, biceps femoris) but was normal elsewhere (Supplementary Table S1). A final diagnosis of sensory neuronopathy (Camdessanché score = 6.5) associated with left sciatic mononeuropathy was made.

We excluded genetic variants in *PMP22* and *RFC1* genes. We excluded all known genetic cause of sensory neuropathy and performed exome sequencing on the three patients and one asymptomatic relative (Supplementary methods). After filtering exome sequencing evidenced 16 rare variants shared by the three patients and absent in the unaffected uncle (Supplementary Table S2). Only a missense variant in *KLHL3* (NM_017415.3: c.1582C > T, p.(Arg528Cys)) in exon 13 could explain the phenotype. This variant has been described as being pathogenic [3,4], located in a mutational hotspot [3] and is present with an extremely low frequency in the gnomAD database (gnomAD v4.0.0 = 1/628744). Another variant NM_017415.3: c.1583G > A, p.(Arg528His) which affects the same codon but has a different amino acid change has also been reported as pathogenic [3,4],. The *KLHL3* gene has a low rate of benign missense variants. Prediction tools and segregation analyses reinforced the pathogenicity. According to American College of Medical Genetics and Genomics guidelines [5], this variation should be considered as “Pathogenic”. Pedigree and Sanger sequencing are shown in Supplementary Fig. S1.

## 3 Discussion

This is the first description of sensory neuropathy associated with *KLHL3* mutation. Although this is the only family reported today with such clinical presentation, the presence in the same family of sensory neuronopathy, a rare presentation of peripheral neuropathy [6], strongly suggests a genetic disorder. Whereas PHAII in our family is explained by p.(Arg528Cys) mutation in the *KLHL3* gene, as previously reported [3,4], the sensory neuropathy is likely to be a new phenotype associated with *KLHL3*, considering the extensive genetic screening, our segregation analysis and that the variant has been reported to be pathogenic. Our study expands the genotype-phenotype spectrum of *KLHL3* with the addition of sensory neuronopathy. We propose to screen *KLHL3* gene in patients presenting with both PHAII and sensory neuropathy symptoms. Moreover, *KLHL3* gene should be included in genetic explorations of inherited sensory neuropathies.

Although there were no other candidate genes in our patients for sensory neuropathy, we cannot exclude that we missed a variant located in a deep intronic region or a gene associated with a hereditary neuropathy that was not yet reported before in the literature. Additional functional studies may be relevant to demonstrate the role of *KLHL3* in this family. We noted phenotypic heterogeneity of sensory neuropathy. The observed disability differs from a severe and early handicap for the proband to a paucisymptomatic impact for her mother and grandfather. Different genetic modifiers or environmental factors may influence on the variable expression of sensory neuropathy in the family. This scarcity of neurological signs in some patients could explain why sensory neuropathy related to *KLHL3* has not been described before. Since neurological signs can be quite subtle, patients with PAHII should be referred to a neurologist and have NCS performed to screen for this condition.

Missenses mutants at position 528 of KLHL3 have been described to impair interaction with WNK1 and WNK4 and to impair ubiquitination of WNK4. In PAHII, this accumulation of WNK1 and WNK4 proteins leads to an activation of sodium and sodium-chloride channels [7]. The mechanism leading to sensory neuropathy in *KLHL3* mutation is not currently understood. One hypothesis would be that alteration of extracellular sodium could alter different channels in sensory nerves, for example, sodium-voltage gated channels, which are highly expressed in nociceptive and sympathetic neurons of the peripheral nervous system, and play a major role in neuropathic pain [8].

## 4 Conclusion

Our study is in favour of an extension of the phenotype associated with *KLHL3* mutation. However, a larger cohort size and further studies are needed to confirm this association between *KLHL3* variants and sensory neuropathy. It may also require additional explorations, such as performing NCS on other *KLHL3* patients. In future research, it may also be relevant to check if *Klhl3* knockout mice (*Klhl3*-/-) [9] display a sensory neuropathy phenotype, and deregulation of sodium-voltage gated channels in the dorsal, root ganglia.

## CRediT authorship contribution statement

**J.B. Davion:** Writing – original draft, Investigation. **I. Coku:** Writing – original draft, Formal analysis, Data curation. **A. Wissocq:** Data curation. **A. Genet:** Data curation. **J. Poupart:** Investigation. **L. Defebvre:** Writing – review & editing. **V. Huin:** Writing – review & editing, Writing – original draft, Supervision, Funding acquisition, Formal analysis, Data curation, Conceptualization.

## Ethics statement

Written informed consent for genetic testing for diagnosis, research, and publication of relevant findings was obtained from all study participants. The study was conducted in accordance with the Declaration of Helsinki. The data supporting the findings of this study are available from the corresponding author upon reasonable request.

## Funding

This work was supported by the “*Institut National de la Santé et de la Recherche Médicale*” (INSERM), the *Université de Lille*, the 10.13039/501100015613Lille University Hospital, the *France Alzheimer* charity (AAP PFA 2021, grant number: 6242) and the Programs d'investissements d'avenir LabEx (excellence laboratory) 10.13039/100015867DISTALZ (Development of Innovative Strategies for a Transdisciplinary approach to ALZheimer's disease).

## Declaration of competing interest

The authors declare that they have no known competing financial interests or personal relationships that could have appeared to influence the work reported in this paper.

## References

[1] Ellison D.H., Adam M.P., Everman D.B., Mirzaa G.M., Pagon R.A., Wallace S.E., Bean L.J., Gripp K.W., Amemiya A. (1993). GeneReviews®.

[2] Camdessanché J.-P., Jousserand G., Ferraud K., Vial C., Petiot P., Honnorat J., Antoine J.-C. (2009). The pattern and diagnostic criteria of sensory neuronopathy: a case–control study. Brain.

[3] Boyden L.M., Choi M., Choate K.A., Nelson-Williams C.J., Farhi A., Toka H.R., Tikhonova I.R., Bjornson R., Mane S.M., Colussi G., Lebel M., Gordon R.D., Semmekrot B.A., Poujol A., Välimäki M.J., De Ferrari M.E., Sanjad S.A., Gutkin M., Karet F.E., Tucci J.R., Stockigt J.R., Keppler-Noreuil K.M., Porter C.C., Anand S.K., Whiteford M.L., Davis I.D., Dewar S.B., Bettinelli A., Fadrowski J.J., Belsha C.W., Hunley T.E., Nelson R.D., Trachtman H., Cole T.R.P., Pinsk M., Bockenhauer D., Shenoy M., Vaidyanathan P., Foreman J.W., Rasoulpour M., Thameem F., Al-Shahrouri H.Z., Radhakrishnan J., Gharavi A.G., Goilav B., Lifton R.P. (2012). Mutations in kelch-like 3 and cullin 3 cause hypertension and electrolyte abnormalities. Nature.

[4] Louis-Dit-Picard H., Barc J., Trujillano D., Miserey-Lenkei S., Bouatia-Naji N., Pylypenko O., Beaurain G., Bonnefond A., Sand O., Simian C., Vidal-Petiot E., Soukaseum C., Mandet C., Broux F., Chabre O., Delahousse M., Esnault V., Fiquet B., Houillier P., Bagnis C.I., Koenig J., Konrad M., Landais P., Mourani C., Niaudet P., Probst V., Thauvin C., Unwin R.J., Soroka S.D., Ehret G., Ossowski S., Caulfield M., Bruneval P., Estivill X., Froguel P., Hadchouel J., Schott J.-J., Jeunemaitre X. (2012). KLHL3 mutations cause familial hyperkalemic hypertension by impairing ion transport in the distal nephron. Nat. Genet..

[5] Richards S., Aziz N., Bale S., Bick D., Das S., Gastier-Foster J., Grody W.W., Hegde M., Lyon E., Spector E., Voelkerding K., Rehm H.L. (2015). Standards and guidelines for the interpretation of sequence variants: a joint consensus recommendation of the American College of medical genetics and Genomics and the association for molecular pathology. Genet. Med..

[6] Fargeot G., Echaniz-Laguna A. (2021). Sensory neuronopathies: new genes, new antibodies and new concepts. J. Neurol. Neurosurg. Psychiatry.

[7] Susa K., Sohara E., Rai T., Zeniya M., Mori Y., Mori T., Chiga M., Nomura N., Nishida H., Takahashi D., Isobe K., Inoue Y., Takeishi K., Takeda N., Sasaki S., Uchida S. (2014). Impaired degradation of WNK1 and WNK4 kinases causes PHAII in mutant KLHL3 knock-in mice. Hum. Mol. Genet..

[8] Bennett D.L., Clark A.J., Huang J., Waxman S.G., Dib-Hajj S.D. (2019). The role of voltage-gated sodium channels in pain signaling. Physiol. Rev..

[9] Sasaki E., Susa K., Mori T., Isobe K., Araki Y., Inoue Y., Yoshizaki Y., Ando F., Mori Y., Mandai S., Zeniya M., Takahashi D., Nomura N., Rai T., Uchida S., Sohara E. (2017). *KLHL3* knockout mice reveal the physiological role of KLHL3 and the pathophysiology of pseudohypoaldosteronism type II caused by mutant KLHL3. Mol. Cell Biol..

