# Supplementary material for: Pseudohypoaldosteronism type II and sensory neuropathy associated with a heterozygous pathogenic variant in KLHL3 gene, a case report
Source: Heliyon. 2024 Oct 29;10(21):e39891. doi: 10.1016/j.heliyon.2024.e39891 (PMC11566686; doi:10.1016/j.heliyon.2024.e39891)
Supplement: Multimedia component 1 [file mmc1.docx]

TITLE OF THE ARTICLE:

Pseudohypoaldosteronism type II and sensory neuropathy associated with a heterozygous pathogenic variant in KLHL3 gene, a case report

AUTHORS:

Davion JB^1,2†^, Coku I^1†^, Wissocq A^3^, Genet A^3^, Poupart J^4^, Defebvre L^1,5^, and Huin V^1,3,*^

AFFILIATIONS:

^1^ Univ. Lille, Inserm, CHU Lille, U1172 - LilNCog (JPARC) - Lille Neuroscience and Cognition, F-59000 Lille, France

^2^ Centre de Référence des Maladies Neuromusculaires, CHU Lille, Hôpital Pierre Swynghedauw, F-59000 Lille, France

^3^ CHU Lille, Toxicology and Genopathies Department, UF Neurobiology, F-59000 Lille, France

^4^ Lille Catholic Hospital (GHICL), Neurology Department, F-59462 Lomme, France

^5^ CHU Lille, Neurology and Movement Disorders Department, F-59000, Lille

^†^ These authors contributed equally to this work

* Corresponding author

CORRESPONDING AUTHOR DETAILS:

Dr. Vincent Huin

[vincent.huin@chru-lille.fr](mailto:vincent.huin@chru-lille.frvincent.huin@inserm.fr)

[vincent.huin@inserm.fr](mailto:vincent.huin@chru-lille.frvincent.huin@inserm.fr)

Telephone: +33 (0)3 20 44 48 01

Fax: +33 (0)3 20 44 49 57

SUPPLEMENTARY METHODS:

***Genetic screening of frequent inherited sensory neuropathy***

Genomic DNA was isolated from peripheral blood according to standard procedures. We performed multiplex ligation-dependent probe amplification on all patients using SALSA MLPA Probe mixes P033 for the molecular diagnosis of *PMP22* copy number variation (MRC Holland, Amsterdam, The Netherlands) according to the manufacturer's instructions.

The identification of biallelic (AAGGG)_n_ expansions in *RFC1* included for all patients a fluorescent duplex PCR, three repeat primer-PCRs targeting respectively the motifs (AAAAG)_n_, (AAAGG)_n_ and (AAGGG)_n_ and LR-PCR with Southern-blotting of the PCR product. The length of the PCR products was analyzed by capillary electrophoresis on an ABI3730 DNA analyzer (Applied Biosystems, Foster City, CA, USA).

***Whole exome study***

*Bioinformatics analysis*

We performed exome sequencing on the three patients and one asymptomatic relative using a NovaSeq6000™ instrument and DRAGEN™ software (Illumina, San Diego, California, USA). Exon capture and libraries was prepared using the TrueSeq DNA Exome S4 kit (Illumina, San Diego, CA, USA) according to the manufacturer’s instructions. Pooled libraries were sequenced using paired-end, 150-cycle chemistry on an Illumina NovaSeq6000 (Illumina, San Diego, CA, USA).

The sequence data were aligned to the hg38 assembly version of the human genome using BWA v0.7.17. Variant calling, joint genotyping, and recalibration were performed using GATK v3.7. Variant annotation was performed using Variant Effect Predictor (https://www.ensembl.org/info/docs/tools/vep/index.html) and Dragen software (Illumina, San Diego, CA, USA). Harmful effects from SIFT, Polyphen2 HumDiv, Polyphen2 HumVar, FATHMM, AlphaMissense, REVEL, ClinPred, Meta SVM, Meta LR, Mistic were obtained using Variant Effect Predictor (https://www.ensembl.org/info/docs/tools/vep/index.html). We used DRAGEN CNV Baseline Builder (Illumina, San Diego, CA, USA) to analyze structural variations.

*Variant filtering*

During the search of candidate genes, we used the following filters:

-For all variants: (i) quality filter (PASS GATK filter, variant coverage ≥10), (ii) effect on coding sequence (exonic non-synonymous or canonical splice variant).

-For variants compatible with dominant inheritance: (i) allelic frequency between 0.3 and 0.7, (ii) frequency in the public databases: we considered only those with an allele count < 5 in the gnomAD v2 and v3 databases [http://gnomad.broadinstitute.org/].

-For variants compatible with recessive inheritance (homozygous variants or two heterozygous variants in the same gene): (i) allelic frequency between 0.9 and 1, (ii) frequency in the public databases: we considered only those with an allele count < 3 at the homozygous/hemizygous state in the gnomAD v2 and v3 databases [http://gnomad.broadinstitute.org/].

-For the structural variant analysis, we used the following filters: (i) quality filter (PASS DRAGEN CNV filter), (ii) frequency in the public databases.

Before the whole genes analysis, the following 154 hereditary neuropathy associated genes were first analyzed: *AARS1*, *ABHD12*, *ADCY6*, *ADGRG6*, *AIFM1*, *ARHGEF10*, *ATL1*, *ATL3*, *ATP1A1*, *ATP7A*, *BAG3*, *BICD2*, *BSCL2*, *CCT5*, *CHCHD10*, *CLTCL1*, *CNTNAP1*, *COA7*, *COX6A1*, *CTDP1*, *DARS2*, *DCAF8*, *DCTN1*, *DCTN2*, *DGAT1*, *DGAT2*, *DHH*, *DHTKD1*, *DNAJB2*, *DNM2*, *DNMT1*, *DRP2*, *DST*, *DYNC1H1*, *ECEL1*, *EGR2*, *ELP1*, *FBLN5*, *FBXO38*, *FGD4*, *FIG4*, *FLVCR1*, *FXN*, *GAN*, *GARS1*, *GDAP1*, *GDNF*, *GJB1*, *GJB3*, *GLA*, *GLDN*, *GNB4*, *HADHB*, *HARS1*, *HINT1*, *HK1*, *HOOK1*, *HOXD10*, *HSPB1*, *HSPB3*, *HSPB8*, *IARS2*, *IFRD1*, *IGHMBP2*, *INF2*, *JPH1*, *KARS1*, *KCNA4*, *KIF1A*, *KIF1B*, *KIF5A*, *LGI4*, *LITAF*, *LMNA*, *LRSAM1*, *MARS1*, *MED25*, *MED9*, *MFN2*, *MICAL1*, *MME*, *MORC2*, *MPV17*, *MPZ*, *MTMR2*, *MTRFR*, *MYH14*, *NAGLU*, *NAT8*, *NDRG1*, *NEFH*, *NEFL*, *NGF*, *NTRK1*, *OPA1*, *PDK3*, *PEX7*, *PHYH*, *PLEKHG5*, *PMP2*, *PMP22*, *PNKP*, *POLG*, *PRDM12*, *PRDX6*, *PRPH*, *PRPS1*, *PRX*, *PXMP2*, *RAB7A*, *REEP1*, *RETREG1*, *RNF170*, *SACS*, *SBF1*, *SBF2*, *SCN10A*, *SCN11A*, *SCN9A*, *SEPTIN9*, *SETX*, *SH3TC2*, *SIGMAR1*, *SLC12A6*, *SLC25A19*, *SLC25A46*, *SLC5A7*, *SORD*, *SOX10*, *SPG11*, *SPTBN4*, *SPTLC1*, *SPTLC2*, *SPTLC3*, *SURF1*, *SYNE1*, *SYT2*, *TDP1*, *TECPR2*, *TFG*, *TRIM2*, *TRPA1*, *TRPV3*, *TRPV4*, *TTR*, *TWNK*, *UBQLN2*, *UNC50*, *VAPB*, *VCP*, *VRK1*, *WARS1*, *WNK1*, *YARS1.*

In the 154 hereditary neuropathy associated genes we look for any variant with high (nonsenses, mutations of the canonical splice sites, start loss, stop loss, insertions/deletions resulting in a frameshift, large deletions/insertions), moderate (missenses, insertions/deletions with preservation of the reading frame) or low impact (synonymous variants, other splice variants).

Thereafter, we analyzed all the variants with high or moderate impact in the remaining genes from the whole exomes.

Splice effect was analysed using SpliceAI and AbSplice. Gene expression was analysed with GTEx Portal (<https://www.gtexportal.org/home/>). We considered a median expression < 1 transcript per million in the tibial nerve to define genes with poor expression in in peripheral nerves.

*Segregation analyses*

We excluded rare variants that were not shared rare variants between the three affected patients in the family and/or present in the unaffected index case’s uncle. Results of the filtering and of the segregation analyses are shown in the supplementary Table S2.

SUPPLEMENTARY FIGURE:

**Supplementary figure S1. Pedigrees and genetic variant of the family.**

SUPPLEMENTARY TABLES:

### **Supplementary Table S1. Nerve conduction studies and needle electromyography of the three patients.**

Distal latencies are expressed in milliseconds, sensory amplitudes in microvolts, motor amplitudes in millivolts, and conduction speed in motos/second. NCS: nerve conduction studies, DL: distal latency, DA: distal amplitude, CS: conduction speed, EMG: electromyography, L: Left; R: right, EDB: extensor digitorum brevis, APB: abductor hallucis brevis, APB: abductor pollicis brevis

|  |  | Proband | | | Mother | | | Maternal grandfather | | |
| --- | --- | --- | --- | --- | --- | --- | --- | --- | --- | --- |
|  |  | DL | DA | CS | DL | DA | CS | DL | DA | CS |
| Sensory NCS | Sural L | - | 0 | - | 1.9 | **7.3** | 47 | 1.7 | **3.3** | 47 |
|  | Sural R | 1.7 | **3.8** | 38 | 1.9 | **7.6** | 48 | 2.0 | **3.1** | 39 |
|  | Fibular L | NA | NA | NA | - | **0** | - | - | **0** | - |
|  | Fibular R | NA | NA | NA | - | **0** | - | - | **0** | - |
|  | Median L | 2.4 | **13.8** | 51 | 3.5 | 9.0 | 39 | 3.5 | **7.2** | **43** |
|  | Median R | 2.5 | **9.9** | 50 | 4.7 | 3.4 | 29 | 3.0 | **5.5** | 48 |
|  | Ulnar L | NA | NA | NA | 1.9 | 16.7 | 60 | 2.8 | **5.9** | **43** |
|  | Ulnar R | NA | NA | NA | 2.1 | 17.9 | 55 | 2.3 | **4.1** | 50 |
|  | Radial L | NA | NA | NA | 1.6 | 20.5 | NA | NA | NA | NA |
|  | Radial R | 1.2 | **8.3** | NA | 1.8 | 21.2 | NA | NA | NA | NA |
| Motor NCS | Fibular L (EDB) | 3.4 | 4.6 | 44 | - | **0** | - | - | **0** | - |
|  | Fibular R (EDB) | 3.5 | 4.5 | 44 | 4.0 | 9.1 | 48 | 4.6 | 2.7 | 46 |
|  | Fibular L (TA) | NA | NA | NA | 2.0 | 5.2 | 58 | 2.4 | **0.8** | NA |
|  | Fibular R (TA) | NA | NA | NA | 1.9 | 7.1 | 69 | 2.6 | 6.6 | 49 |
|  | Tibial L (AHB) | 4.6 | 10.5 | NA | NA | NA | NA | - | **0** | - |
|  | Tibial R (AHB) | 3.2 | 12.0 | NA | NA | NA | NA | 4.1 | 8.0 | 40 |
|  | Median L (APB) | 3.2 | 9.4 | 57 | 3.7 | 13.7 | 58 | 4.3 | 7.3 | 55 |
|  | Median R (APB) | 2.8 | 8.8 | 58 | **4.9** | 14.4 | 61 | 4.1 | 6.4 | 52 |

### **Supplementary Table S2: List of the rare genetic variants shared by the three affected patients and absent in the unaffected relative.**

HET: heterozygous; HEM: hemizygous; AD: autosomal dominant; AR: autosomal recessive; XL: X-linked; na: not applicable.

| **Genomic location (hg38)** | **Gene** | **Transcript** | **Variant** | **Status** | **Inheritance in OMIM** | **ACMG classificartion** | **Comments** |
| --- | --- | --- | --- | --- | --- | --- | --- |
| chr2:g.26254463G>A | *HADHB* | NM_000183.3 | c.98G>A, p.(Arg33Gln) | HET | AR | Uncertain significance | Incompatible with a dominant inheritance. |
| chr12:g.48778150G>A | *ADCY6* | NM_015270.5 | c.972C>T, p.(Tyr324=) | HET | AR | Likely benign | Incompatible with a dominant inheritance. No predicted effect on splicing according to SpliceAI and AbSplice. |
| chr12:g.753575_753576delinsTT | *WNK1* | NM_18979.4 | c.10_11delinsTT, p.(Gly4Val) | HET | (AD), AR | Uncertain significance | The phenotypes encompassing neuropathy are only observed with autosomal reccessive inheritance. |
| **chr5:g.137628306G>A** | ***KLHL3*** | **NM_017415.3** | **c.1582C>T, p.(Arg528Cys)** | **HET** | **AD, AR** | **Pathogenic** |  |
| chr3:g.195787991G>C | *MUC4* | NM_018406.7 | c.3589C>G, p.(His1197Asp) | HET | na | Uncertain significance | Poorly expressed in peripheral nerves. |
| chr3:g.195788002G>A | *MUC4* | NM_018406.7 | c.3578C>T, p.(Ala1193Val) | HET | na | Uncertain significance | Poorly expressed in peripheral nerves. |
| chr3:g.195788011G>A | *MUC4* | NM_018406.7 | c.3569C>T, p.(Pro1190Leu) | HET | na | Uncertain significance | Poorly expressed in peripheral nerves. |
| chr7:g.130192632C>G | *TMEM209* | NM_032842.4 | c.765G>C, p.(Arg255Ser) | HET | na | Uncertain significance |  |
| chr7:g.142005531A>G | *MGAM* | NM_001365693.1 | c.1A>G, p.(Met1?) | HET | na | Uncertain significance | **G**ene tolerant to loss of function variants, poorly expressed in peripheral nerves. |
| chr11:g.68690955G>A | *GAL* | NM_015973.5 | c.340G>A, p.(Ala114Thr) | HET | AD | Uncertain significance | Phenotype not compatible, poorly expressed in peripheral nerves. |
| chr11:g.70433697G>A | *CTTN* | NM_005231.4 | c.1495G>A, p.(Ala499Thr) | HET | na | Uncertain significance |  |
| chr11:g.96091959A>G | *MAML2* | NM_032427.4 | c.2072T>C, p.(Leu691Pro) | HET | na | Uncertain significance |  |
| chr17:g.48059405C>G | *NFE2L1* | NM_003204.3 | c.2083C>G, p.(Arg695Gly) | HET | na | Uncertain significance |  |
| chr20:g.45815468C>T | *UBE2C* | NM_001281741.2 | c.208C>T, p.(Leu70Phe) | HET | na | Uncertain significance | Poorly expressed in peripheral nerves. |
| chr20:g.53253682C>T | *TSHZ2* | NM_173485.6 | c.224C>T, p.(Ser75Phe) | HET | na | Uncertain significance |  |
| chrX:g.111682226A>G | *ALG13* | NM_001257231.1 | c.1A>G, p.(Met1?) | HEM/HET | XL | Uncertain significance | Phenotype not compatible. |
